# Leveraging Electron Beam-Inactivated Multi-Strain *Staphylococcus* Vaccine for Preventing BCO Lameness in Broiler Chickens

**DOI:** 10.3390/vaccines13090946

**Published:** 2025-09-04

**Authors:** Ruvindu Perera, Andi Asnayanti, Khawla S. Alharbi, Anh Do, Manel Ben Larbi, Amanda P. Anthney, Anna L. F. V. Assumpcao, Komala Arsi, Geetha Kumar-Phillips, Jossie M. Santamaria, Gisela F. Erf, Tanmaie Kalapala, Suresh D. Pillai, Palmy Jesudhasan, Adnan A. K. Alrubaye

**Affiliations:** 1 Cell and Molecular Biology Program, University of Arkansas, Fayetteville, AR 72701, USA; rperera@uark.edu (R.P.); ka030@uark.edu (K.S.A.); ad086@uark.edu (A.D.); 2 Center of Excellence for Poultry Science, University of Arkansas System, Division of Agriculture, Fayetteville, AR 72701, USA; aasnayan@uark.edu (A.A.); apanthne@uark.edu (A.P.A.); facchettivin@uada.edu (A.L.F.V.A.); gskumar@uark.edu (G.K.-P.); jmsantam@uark.edu (J.M.S.); gferf@uark.edu (G.F.E.); tanmaiek@uark.edu (T.K.); 3 Research Unit of Biodiversity and Resource Development in Mountain Areas of Tunisia (UR17AGR14), Higher School of Agriculture of Mateur, University of Carthage, Mateur 7030, Tunisia; manel.benlarbi@gmail.com; 4 Poultry Production and Product Safety Research Unit, ARS, USDA, Fayetteville, AR 72791, USA; komala.arsi@usda.gov; 5 National Center for Electron Beam Research, Texas A&M University, College Station, TX 77845, USA; suresh.pillai@ag.tamu.edu

**Keywords:** electron beam, *Staphylococcus*, Chondronecrosis, Osteomyelitis, lameness, broilers

## Abstract

Background: Bacterial Chondronecrosis with Osteomyelitis (BCO) is a significant issue affecting the welfare and economy of the broiler industry, causing substantial revenue losses annually. This disease is frequently associated with *Staphylococcus* spp. and *Enterococcus* spp. infections and necrosis of leg and vertebral bones. The typical annual lameness incidence of approximately 3–5% may increase to 30% during outbreaks. Neither the etiology or pathogenesis of the disease has been comprehended, nor have effective preventative measures been identified. Electron beam (eBeam) technology is renowned for producing efficient whole-cell vaccines by preventing bacterial multiplication through irreversible DNA shredding while preserving the integrity of membrane proteins (immunogenic epitopes). This study aims to reduce BCO-induced lameness in broiler chickens via in ovo immunization using eBeam-inactivated multi-strain *Staphylococcus*. Methods: A total 1080 birds were assigned to four vaccination groups: eBeam-inactivated, formalin-inactivated, combination of eBeam- and formalin-inactivated, and sham (vehicle). The birds were directly exposed to aerosolized, natural BCO challenge until 56 days of age. Results: Birds vaccinated with the eBeam-inactivated *Staphylococcus* vaccine showed a significant reduction (>50%) in daily cumulative lameness compared to other groups and a decrease in Staphylococcus colonization was observed in the leg joints of treated birds. Conclusions: the eBeam-inactivated *Staphylococcus* vaccine successfully prevented BCO lameness in broiler chickens.

## 1. Introduction

Bacterial Chondronecrosis with Osteomyelitis (BCO) lameness is a metabolic disease that currently poses a significant issue for animal welfare in the poultry industry, resulting in hundreds of millions of USD in lost revenue globally. Approximately 1 billion broilers out of the global production of 74 billion [1] are produced in Arkansas alone, valued at a total of USD 2.82 billion annually [2]. Annually, up to 5% BCO-related animal losses are reported globally [3]. Nevertheless, dramatic increases in lameness, ranging from 15% [4,5] to 30% [3,6,7] of flocks, have been reported worldwide during episodic outbreaks. The disease affects broilers with heavy body weight, causing discomfort and inability to walk, leading to increased mortality and economic losses from condemnation at marketing age [3]. Based on the currently accepted model of BCO pathogenesis, the heavy weight gain of broiler chickens causes initial mechanical damage to the bone joints. This is accompanied by the colonization and proliferation of opportunistic pathogens in the gastrointestinal (GI) and respiratory tracts, together with weakened immunity and damaged tight junction integrity resulting from stress. BCO subsequently progresses with bacterial translocation into the bloodstream via compromised tight junctions in the GI and respiratory tracts, which follows the subsequent hematogenous distribution of bacteria into pre-existing osteochondrotic microfractures within mechanically stressed bone growth plates [3,8,9,10,11]. Necrosis and inflammation of cartilage and joints of long leg bones exacerbate the persistent infection, eventually manifesting as macroscopic bone degeneration, particularly in the proximal heads of the femurs and tibiae, resulting in visible lameness [10,12].

Despite research on possible interventions, such as probiotics, prebiotics, organic trace minerals, and other feed supplements to alleviate the incidence of BCO lameness in broiler chickens [10,12,13,14,15,16,17], none of the interventions were fully successful in preventing the condition. A previous practice of supplementing poultry feed and water with antibiotics for over forty years [18] was discontinued due to the spread of antibiotic resistance in pathogenic bacteria [19]. Hence, finding an efficient alternative for BCO prevention is essential, which necessitates a competent model of BCO challenge and induction in broiler chickens to evaluate various treatment effects on BCO reduction. The present study employed the aerosol transmission model of BCO induction, developed by our lab [20]. This model features two wire-floor pens placed in front of all other litter-floor pens, positioned closer to the cooling pads (upwind) and separated from the litter-floor pens by a buffer zone that prevents contact. Tunnel fans are placed at the rear end (downwind) of the house. Birds raised on wire floors are continuously stressed by having to walk for feed and water, which are placed at the opposite ends of the pens, making them prone to developing microfractures in leg bones, leading to accelerated lameness. These birds exhale the BCO pathogens into the air, and contaminated aerosol particles are drawn backward by the tunnel fans, infecting the birds raised in litter-floored pens. This model mimics the natural infection in a real-world scenario, as in commercial broiler chicken houses.

*Staphylococcus* spp. is an abundantly isolated genus of bacteria from femoral and tibial BCO lesions of broiler chicken [11,12,20] and hence known to be associated with the pathogenesis of BCO [21,22,23,24]. Controlling *Staphylococcus* infections is challenging as they have evolved several mechanisms to evade immune responses of various hosts. These include adaptations to prevent opsonization [25], phagocytosis [26], and the production of superantigens [27,28]. Whole-cell vaccines are an efficacious means of treating microbial diseases, offering several advantages over other vaccine types, including their relative time-conservation and cost-effectiveness in production, as well as being carriers of multiple immunogenic epitopes [29]. Notably, live whole-cell vaccines mimic natural infections and immune responses, but their drawbacks include reduced stability and the potential for pathogen revival. Inactivated whole-cell vaccines, typically treated with formaldehyde or other alkylating reagents [30], excel in terms of host safety and high stability. However, inactivating agents can cause damage to immunogenic epitopes by forming cross-links of surface proteins and triggering other chemical reactions, resulting in limited immunogenicity and reduced cross-presentation of antigens through the priming of CD8+ T cells [30,31,32,33]. Interestingly, electron beam (eBeam), an ionizing radiation technology, presents a promising alternative for producing inactivated whole-cell vaccines without damaging cell surface proteins (epitopes) [18,34], while achieving irreversible reproductive death of the treated bacteria [35]. This success lies in eBeam’s property of irreparable shredding of the bacterial genome upon exposure (direct effect), coupled with short-lived free-radical attack owing to the products of radiolysis of intracellular solvents (indirect effects) [35,36]. Several studies have demonstrated eBeam’s ability to retain pathogen membrane integrity using fluorescence microscopy and electron microscopy [18,34,37,38]. Praveen et al. (2021) [38] have demonstrated that eBeam-inactivated *Salmonella* Typhimurium (ST) is metabolically active and stimulates upregulation of CD40, MHC-II, CD80, and CD86 in mice immune cells, at similar levels to live ST vaccines, concluding that an eBeam vaccine is as efficient as a live vaccine but as safe as a killed vaccine. eBeam is also approved by the US Food and Drug Administration to inactivate pathogens and spoilage microorganisms in food [39]. eBeam excels above other types of ionizing radiation like gamma and X-rays in terms of its (1) safety and eco-friendliness, (2) cost-effectiveness, (3) high efficiency, (4) producing substantial doses of radiation in few seconds, (5) ability to produce planar, homogenous radiation profiles, and (6) ability to switch on and off [35,40,41,42]. eBeam appears to provide a promising alternative for the efficient preparation of whole-cell vaccines against pathogenic bacteria.

Based on eBeam’s exceptional ability to produce safe and efficient whole-cell-inactivated vaccines and the dominance of *Staphylococcus* in the pathogenicity of BCO, it was hypothesized that efficient control of *Staphylococcus* in broiler chickens would lead to a reduction in lameness. Accordingly, the present study utilized an eBeam-inactivated vaccine containing multiple *Staphylococcus* species to reduce BCO lameness in broiler chickens, significantly. The rationale behind this study is that a well-optimized eBeam would contribute to generate an efficiently inactivated *Staphylococcus* vaccine with unaltered epitopes, which in turn would generate an effective, targeted immune response against *Staphylococcus* in the vaccinated broiler chickens. Thereby, this immune response would eventually contribute to the prevention of BCO lameness via eliminating *Staphylococcus*, prior to causing infection. This study was conducted as a follow-up to a previous study in our research laboratory, which tested the efficacy of BCO reduction in broilers using an eBeam-inactivated vaccine containing two species of *Staphylococcus* [43]. The present study utilized a modified version of the vaccine with dose optimization and incorporation of additional strains of *Staphylococcus*, which are frequently isolated from BCO lesions of lame broilers. Moreover, birds of the previous vaccine study were only challenged with bacterial strains included in the vaccine, but the current study closely mimicked a real-world setting, where the birds were challenged with the natural intestinal and environmental microbiota. The objective of this study is to improve animal welfare and increase revenue via lameness prevention in broiler chickens. In addition, our eBeam vaccine could prevent foodborne diseases related to the consumption of poultry products contaminated with *Staphylococcus* spp. [44,45], thereby ensuring consumer health and well-being.

## 2. Materials and Methods

### 2.1. Bacterial Culture and Vaccine Preparation

Field-isolated strains of *Staphylococcus agnetis*, *Staphylococcus aureus*, and *Staphylococcus lentus* and two strains of *Staphylococcus cohnii* were used. The five strains were inoculated separately in 100 mL of Tryptic Soy Broth (TSB, Difco, Becton Dickinson, Franklin Lakes, NJ, USA) and incubated at 37 °C overnight. The above cultures were pooled on the following day and centrifuged to remove the spent media. The bacterial pellet was resuspended in TSB at a concentration of ~1 × 10^8^ CFU/mL. The above mix of strains was processed as follows to prepare the types of vaccines mentioned below.

#### 2.1.1. Electron Beam (eBeam) Vaccine

The eBeam treatment was conducted at the National Center for Electron Beam Research (NCEBR) at Texas A&M University (TAMU), College Station, Texas. Cocktails of *Staphylococcus* strains at ~1 × 10^8^ CFU/mL in TSB were placed in plastic bags and heat-sealed to prevent leakage, for shipping in accordance with USDA-APHIS regulations. The sealed bags, each containing 20 mL of culture, were placed in triplicate into a second plastic bag and heat-sealed. Each secondary bag was sealed in a leak-proof 95 kPa specimen transport envelope. After each sealing step, the bags were sprayed with 70% Ethanol and dried with clean paper towels. The triple-bagged samples were finally packed in a Department of Transportation- and IATA-approved box for NCEBR, TAMU. A high-energy (10 MeV, 15 kW) linear accelerator was used for the eBeam treatment. Alanine pellet dosimeters, placed on the top and bottom of each sample (triple-bagged *Staphylococcus* mix), were used to confirm the absorbed dose by the samples. The dose-uniformity ratio was maintained as close as possible to 1.0 to ensure that the entire sample received the desired dose. The samples exposed to 8 kiloGrays (kGy) were stored at 4 °C upon receipt and used as the eBeam vaccine.

#### 2.1.2. Formalin-Inactivated (FK) Vaccine

A previously described protocol by Kaminski et al., (2014) [46] was adopted to prepare the FK vaccine. Briefly, 100 mL of the overnight-grown *Staphylococcus* strain cocktail in TSB at ~1 × 10^8^ CFU/mL was treated with 0.6% of formaldehyde (*v*/*v*) (Electron Microscopy Sciences, Hatfield, PA). This solution was incubated at room temperature for 48 h, while stirring at 200 RPM (PC-420D Digital Hot Plate Stirrer, Corning Inc., Corning, NY, USA). Afterwards, the vaccine solution was centrifuged at 1932× *g* for 15 min at 4 °C. The supernatant was discarded, and the cell suspension was resuspended in an equal volume of Phosphate-Buffered Saline (PBS) and centrifuged at 1932× *g* for 15 min at 4 °C. The final bacterial cell pellet was then resuspended in an equal volume of fresh TSB before storage at 4 °C.

#### 2.1.3. Combination (F+E) Vaccine

Equal proportions of both the eBeam- and formalin-inactivated vaccines were mixed to prepare a vaccine that contained both the eBeam- and formalin-inactivated *Staphylococcus* cocktails.

### 2.2. Confirmation of Bacterial Inactivation, Membrane Integrity, and Viability

#### 2.2.1. Bacterial Inactivation

For confirmation of complete bacterial inactivation, eBeam- and formalin-inactivated treated cells were inoculated into TSB. Furthermore, treated samples were serially diluted in Butterfield’s Phosphate Diluent (BPD) [47] and plated onto Mannitol Salt Agar (MSA, Difco, Becton Dickinson) plates. A set of samples inoculated in TSB and plated in MSA plates were incubated overnight at 37 °C, while the remaining samples inoculated in TSB were incubated overnight at room temperature. The above tubes and plates were then incubated at room temperature for a week prior to vaccine administration. Subsequently, the TSB tubes were monitored for bacterial resuscitation for up to a month post-treatment (eBeam/formalin).

#### 2.2.2. Bacterial Membrane Integrity

For evaluating bacterial membrane integrity, the LIVE/DEAD BacLight Bacterial Viability Kit (Invitrogen Inc., Waltham, MA, USA) was used according to the manufacturer’s guidelines. Briefly, bacterial cells (untreated, eBeam-treated, and formalin-treated) were diluted at 1:10 in BPD. Then, the cells were stained with a mixture of SYTO 9 + propidium iodide and incubated in the dark for 15 min at room temperature. Finally, stained bacterial cell samples were evaluated by fluorescence microscopy (BZ-X800 fluorescence microscope, Keyence, Osaka, Japan) in a 12-well, flat-bottom glass plate (Corning Inc., Kennebunk, ME, USA).

#### 2.2.3. Bacterial Cell Viability

To support the selection of 8 kGy as the desired eBeam dose for the vaccine, data from a previously conducted viability assay of eBeam- and formalin-inactivated *Staphylococcus aureus* and *Staphylococcus agnetis* were utilized. For this purpose, the BacTiter-Glo™ Microbial Cell Viability Assay (Promega, Madison, WI, USA) was followed according to the manufacturer’s instructions. Shortly, 100 µL of untreated/control (shipped to NCEBR but not eBeam-treated), eBeam-treated (at 8, 9, and 10 kGy), and formalin-treated bacteria of each of the above strains in TSB at ~1 × 10^8^ CFU/mL were added to a 96-well, flat-bottom, dark-walled plate (Invitrogen Inc., Waltham, MA, USA) in triplicate. This was followed by the addition of 100 µL of BacTiter-Glo™ Reagent to each well and incubation at room temperature on a plate shaker (VWR International, Radnor, PA, USA) at 150 RPM for 5 min (covered by an Aluminum foil to protect from the light). Finally, luminescence was recorded using a BioTek Synergy Multi-Mode Microplate Reader (Agilent Technologies, Santa Clara, CA, USA).

### 2.3. Broiler Chicken Vaccination Trial

#### 2.3.1. Egg Placement and in Ovo Vaccination

**Figure 1:**
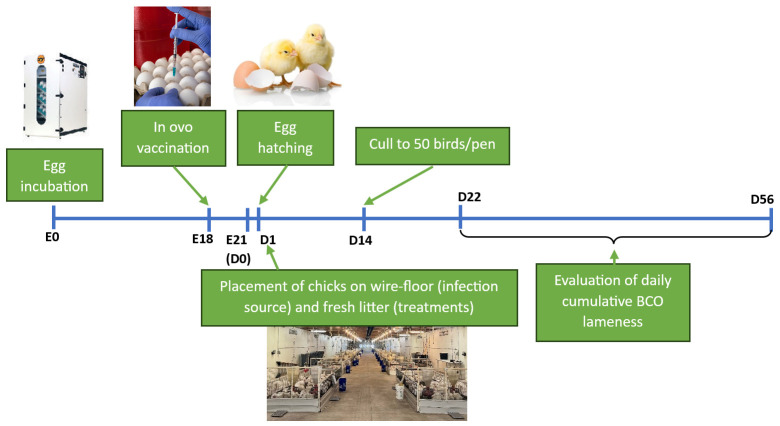
Schematic illustrating the study timeline. E: age of embryos throughout incubation, D: age of birds post-hatching.

All procedures and protocols were approved by the University of Arkansas System, Division of Agriculture, Institutional Animal Care and Use Committee (IACUC), Protocol # 23058. Figure 1 illustrates the summary of this study’s timeline.

One thousand five hundred and forty-three (1543) broiler eggs (0-day-old Cobb500) were obtained from a local hatchery and placed for incubation following IACUC-approved conditions (temperature = 99.6 °F; RH = 85%). They were candled for viability on days 10 and 18 of incubation to remove spoiled, damaged, or non-viable eggs. The in ovo vaccination was administered to the viable eggs on D18 of incubation. The eBeam- and formalin-inactivated vaccines were diluted in fresh TSB to ~1 × 10^7^ CFU/mL. Equal proportions of both vaccines were mixed to prepare the combination vaccine. For vaccination, the large ends of all eggs were sterilized with 70% Ethanol, wiped, and gently pierced with a 0.25″-long 18-gauge needle. The vaccine or sham (TSB) was administered directly into the amniotic cavity using a 1 mL tuberculin syringe with a 1.5″-long, 25-gauge needle equipped with a modified needle guard and injected at an angle to limit all injections to a depth of 3 cm. The different treatment groups of this study are explained in Table 1 below. Following individual injections, injection sites on all eggs were sealed with melted paraffin using cotton swabs. Finally, vaccinated eggs were placed in hatching cabinets for further incubation (temperature = 98 °F; relative humidity = 85%) until hatching.

#### 2.3.2. Live Bird Study and Sampling

**Figure 2:**
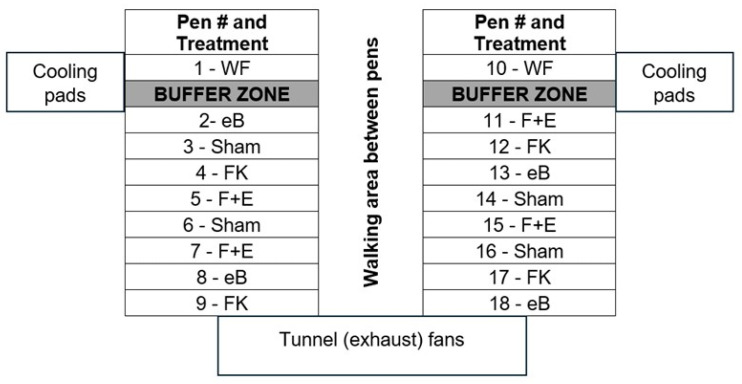
Schematic diagram representing the relative locations of the pens in our facility and the treatment groups of birds in each pen (WF—wire-floor pens with sham-vaccinated birds; eB—litter-floor pens with birds that received the eBeam-inactivated *Staphylococcus* vaccine; FK—litter-floor pens with birds that received the formalin-inactivated *Staphylococcus* vaccine; F+E—litter-floor pens with birds that received the combination *Staphylococcus* vaccine; sham—litter-floor pens with birds that received the sham vaccine). Each pen had 60 birds on the day of hatching.

One thousand and eighty (1080) birds were placed at 60 birds/pen on the day of hatching, according to the design in Figure 2.

Sixteen (16) litter-floor pens and two wire-floor pens of 5′ × 10′ were used following the aerosol transmission infection model developed by our lab [20]. The bird stocking density was maintained at 0.83 ft^2^/bird (D1–14) and 1 ft^2^/bird (D15–56), and the birds of all pens were culled down to 50 on D14. Feed and water were provided ad libitum. Standard crumble starter diet (D1–34) followed by pellet finisher diet (D34–56) were provided. Thermoneutral temperatures were maintained throughout at 32 °C (90 °F) for D1–3, 31 °C (88 °F) for D4–6, 29 °C (85 °F) for D7–10, 26 °C (80 °F) for D11–14, and 24 °C (75 °F) thereafter. A heat lamp was positioned in each pen to provide supplemental heat through D28 when necessary. The photoperiod was 23L:1D, with white light throughout the experiment. All remaining birds were humanely euthanized at the end of the trial on D56.

Starting from D22 of the experiment, birds of each pen were prompted to move daily (using brooms), and any bird that was reluctant to move/stand up was identified as clinically lame [48]. Upon identification as lame, the birds were humanely euthanized and necropsied to expose the proximal femoral and tibial heads, to determine the severity of the progression of bone necrosis. BCO lesion categorization, based on the severity of necrosis, was adopted from Wideman (2016) [3]. On days 16, 32, and 56, 12 birds/treatment (4 birds/pen) were randomly selected and sampled as follows. Blood was collected (ante-mortem) in EDTA vacutainers for the analysis and comparison of plasma *Staphylococcus*-specific antibody (IgM, IgY, and IgA) profiles via Enzyme-Linked Immunosorbent Assay (ELISA) and whole-blood cell populations via immunofluorescent staining followed by flow cytometry (days 16 and 32) among treatments. Approximately 2.5 cm of tracheal tissue was collected without contamination by blood into 1.5 mL BPD to extract tracheal mucus. Later, the lumens of tracheal tissues were flushed with the same BPD solution several times to move the mucus into the BPD prior to discarding the tissue for the analysis and comparison of IgA profiles.

On D56, BCO lesion swabs of 2 bones/bird (femur and tibia) from 4 birds/treatment (1 bird/pen) were collected aseptically and plated onto CHROMagar Orientation (CO, CHROMagar™, Paris, France) and Tryptic Soy Agar (TSA, Difco, Becton Dickinson). The above plates were incubated overnight at 37 °C for bacterial enumeration. The total number of colonies and morphologies were recorded, and the isolated colonies were re-streaked on fresh media plates. Representative colonies were subjected to DNA extraction, PCR amplification of the rDNA 16S V1-V5 region, and sequencing for the identification of BCO-causing bacterial species.

### 2.4. Sample Processing

#### 2.4.1. Bacterial Species Identification from BCO Lesions

Isolated pure cultures of bacteria obtained from BCO lesions, as explained above, were inoculated in TSB and incubated overnight at 37 °C. For each pure culture, 40% glycerol stocks were made for preservation at −80 °C. DNA extraction from the cultures was performed using the NucleoSpin^®^ Microbial DNA kit (Macherey-Nagel, Düren, Germany), following the manufacturer’s instructions. The extracted DNA was then PCR-amplified using ribosomal RNA (rRNA) as a biomarker. Conventional PCR-based amplification of the rDNA 16S V1-V5 region was performed using the protocol described by Asnayanti et al. [49]. Briefly, the PCR mixture of 50 μL was prepared using 25 μL of Phusion^®^ High-Fidelity PCR Master Mix (New England Biolabs^®^ Inc., Massachusetts, USA), 0.5 μM of each forward (5′-AGAGTTTGATCCTGGCTCAG-3′) and reverse (5′-GTGCGGGCCCCCGTCAATTC-3′) primer, 1.5 μL of dimethyl sulphoxide (DMSO), 2 μL of DNA samples, and 20.5 μL of nuclease-free water. A thermal cycler (Bio-Rad T100TM, Hercules, CA, USA) was set to 98 °C for 30 s, 98 °C for 10 s, 71 °C for 30 s, and 72 °C for 30 s (35 cycles), followed by 72 °C for 3 min, and an infinite hold of 4 °C was used for the amplification. Afterwards, 40–60 ng/µL of the amplified DNA, along with 2–10 pmol/µL of the above primers, was shipped to the Eurofins Genomics Lab (Louisville, KY, USA) for sequencing. Sequence results were visualized using the ApE Plasmid Editor software [50] and blasted (using the Blastn tool) against the database of the National Center for Biotechnology Information (NCBI) (https://www.ncbi.nlm.nih.gov/ (accessed on 01/05/2025)). The species were identified from the database with a sequence similarity of greater than 98%.

#### 2.4.2. Analysis and Comparison of Antibody (IgM, IgY, and IgA) Profiles

Relative levels of *Staphylococcus*-specific IgM, IgY, and IgA in blood, as well as mucosal IgA levels, were determined by ELISA. Briefly, a 1 × 10^7^ CFU/mL *Staphylococcus* strain cocktail (comprising the same strains as the vaccine) was suspended in 0.05 M Coating buffer (0.05 M NaHCO_3_, 0.05 M Na_2_CO_3_, pH 7.4). Then, 96-well flat-bottom plates were coated with 100 µL of the inactivated bacterial cell suspension per well. The plates were then incubated at 37 °C for 2 h, followed by an overnight incubation at 4 °C to allow the *Staphylococcus* cells to adhere. Next, the wells of all plates were washed with a washing solution (50 mM Tris, 0.14 M NaCl, 0.05% Tween 20; pH 8.0) three times (leaving the wash solution in the wells for 2 min each cycle). The solutions in the wells were thoroughly aspirated, and the *Staphylococcus*-coated plates were stored at 4 °C for further use. For each assay, the wells of the plates were blocked with 200 µL of blocking buffer [50 mM Tris, 0.14 M NaCl, 1% bovine serum albumin (BSA) (Sigma-Aldrich, St. Louis, MO), pH 8.0] and incubated at room temperature (RT) for 30 min, followed by 3 washes. Frozen tracheal mucus and plasma samples were thawed and diluted (titers determined via pre-tests) in sample diluent buffer (50 mM Tris, 0.14 M NaCl, 1% BSA, 0.05% Tween 20, and pH 8.0) and added in triplicate at 100 µL/well. Sample dilutions for each assay were as follows: IgM: 1/400 (D16), 1/800 (D32), and 1/3200 (D56); IgY: 1/200 (D16 and D32 samples) and 1/600 (D56); IgA: 1/50 (D16 and D32), 1/400 (D56) with tracheal mucus, and 1/50 (D16), 1/100 (D32), 1/200 (D56) with plasma. Aliquots of plasma and tracheal mucus samples were pooled per collection day prior to freezing. Nine doubling dilutions (100 μL/well; 2 wells/dilution) of sample pools were included in all assays as positive controls to generate a standard curve. Furthermore, each plate contained 3 ‘Blank’ wells, containing the highest concentration of the sample pool for each assay, and 3 wells of non-specific binding (NSB) control, containing diluent buffer at 100 µL/well. After adding samples and controls, the plates were incubated at 37 °C for 2 h, followed by 3 washes. Horse-radish peroxidase (HRP)-conjugated, goat anti-chicken IgG (Ig γ-heavy chain), IgM, and IgA detection antibodies (Bethyl Laboratories, Montgomery, TX, USA) were diluted in diluent buffer at 1/10,000, 1/5000, and 1/2500 for the detection of IgY, IgM, and IgA, respectively. Each well received the above detection antibodies (in accordance with the particular assay) at 100 µL/well, except for the ‘Blank’ wells, which received the unconjugated (no HRP enzyme) detection antibody at 100 µL/well. The plates were then incubated for 1 h at 37 °C, followed by 3 washes. Afterwards, 100 µL/well of 3,3′,5,5′-tetramethylbenzidine substrate solution (TMB, Thermofisher, Waltham, MA, USA) was added and incubated at 37 °C for 15 min. Finally, 100 µL/well of 2 M sulfuric acid was added to stop the reaction. Absorbance at 450 nm was measured for each plate using a 96-well spectrophotometer (ELx800, BioTek, Winooski, VT, USA). The absorbance units (AU) for the pooled sample dilutions were used to generate a standard curve for each plate, and the line equation was used to adjust relative a.u. levels of IgY, IgM, and IgA for each assay.

#### 2.4.3. Flow Cytometry

Whole-blood immunofluorescence staining and cell population analysis by flow cytometry were carried out as described [43,51,52]. Briefly, 20 µL of EDTA- blood was diluted in 980 µL of PBS+ staining buffer (PBS pH 7.2 +1% BSA + 0.1% NaN_3_) (1:50). Then, 50 µL of each diluted blood sample was mixed with 50 µL of the respective antibody mixture, as explained below, and incubated at 4 °C for 45 min, protected from light. All antibodies were purchased from Southern Biotech (Birmingham, AL, USA) unless otherwise specified. Three combinations of mouse-anti-chicken monoclonal antibodies were used: (1) FITC-CD41/61 (Biorad, Hercules, CA, USA), PE-CD3, and SPRD-CD45 to identify leucocytes (CD45^+^CD41/61^−^), T cells (CD45^+^CD3^+^), and thrombocytes (CD45^low^CD41/61^+^), (2) APC-CD45, FITC-CD4, PE- γδTCR, and SPRD-CD8α to identify T cell subsets, and (3) FITC-KUL01, PE-Bu1, and SPRD-CD45 to identify monocytes (CD45^+^KUL01^+^Bu-1^−^) and B cells (CD45^+^KUL01^−^Bu-1^+^), respectively. Heterophils were identified based on forward- and side-scatter characteristics of CD45^+^KUL01^−^Bu-1^−^ cells [51]. Afterwards, 150 µL of PBS+ was added to all samples (1:250 final blood dilution). Samples were analyzed using a BD C6 Accuri flow cytometer (Becton Dickinson, San Jose, CA, USA). Flow cytometry data were analyzed using FlowJo software version 10.10.0 (Becton Dickinson).

### 2.5. Statistical Analysis

One-way Analysis of Variance (ANOVA) followed by Tukey’s multiple-mean comparison test was conducted to analyze the luminescence data to compare bacterial viability between treatments on GraphPad Prism software version 10.2.0 (San Diego, CA, USA). Microsoft Excel 365 (Microsoft, Redmond, WA, USA) software was used to analyze the percentage of daily cumulative lameness and the percentage of bacterial isolates from BCO lesions. The impact of treatments on lameness reduction was examined using a logistic regression model, utilizing a Generalized Linear Model in R version 4.2.2 (R Foundation for Statistical Computing, Vienna, Austria). Kruskal–Wallis Tests followed by Dunn post hoc tests, adapted from Yang et al., (2023) [53] were used to compare the total percentages of BCO lesions between treatment groups, separately for femoral and tibial lesions, on JMP Pro software version 18.0.2. Effects of time, treatment, and time-and-treatment interactions on the differences in antibody responses and concentrations of blood leukocyte populations were analyzed using two-way ANOVA followed by Tukey’s multiple-mean comparison test on GraphPad Prism. No further adjustments were made to the multiple comparisons following the post hoc tests. Continuous data were presented as means ± standard errors of the means (SEMs). Statistical significance was considered at *p* values < 0.05.

## 3. Results

### 3.1. eBeam-Treated Staphylococcus Cells Were Inactivated Entirely, While Retaining Their Membrane Integrity and Higher Viability than Formalin-Treated Staphylococcus

The present study is a follow-up to a broiler vaccination trial that used an eBeam-inactivated *Staphylococcus* vaccine (containing *Staphylococcus aureus* and *Staphylococcus agnetis*) conducted by our laboratory, which employed an eBeam dose of 10 kGy [43]. For the present study, it was hypothesized that if complete inactivation could be achieved by a lower eBeam dose, it would be less detrimental to the bacterial cells, which would increase the immunogenicity to the vaccinated birds.

**Figure 3:**
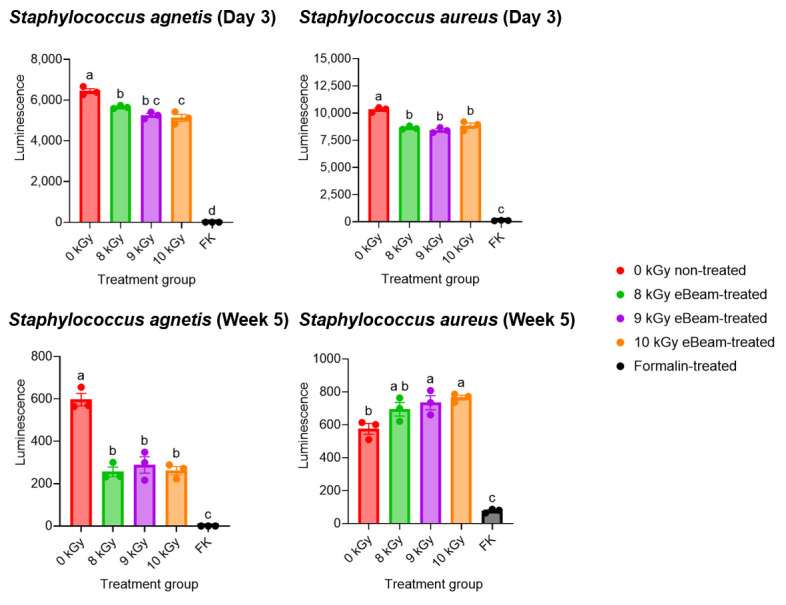
eBeam-treated bacteria retained viability. Comparison of the luminescence (i.e., viability) of non-treated (0 kGy), formalin-inactivated (FK), and eBeam-treated (8, 9 and 10 kGy) *Staphylococcus aureus* and *Staphylococcus agnetis* in the first week (D3) and the fifth week of inactivation. Graphs show means ± SEMs. a–c: Bars with different letters depict significant differences between treatments (*p* < 0.05).

No growth was observed in the TSB tubes as well as the MSA plates inoculated with inactivated bacteria until the point of vaccine administration (within the first week of inactivation) and up to a month after inactivation. These results indicate that both eBeam and formalin were capable of completely inactivating the *Staphylococcus* cocktail, thereby preventing both multiplication and resuscitation. This also confirms the success of the selected eBeam dose for inactivation. Accordingly, the viability was compared across *Staphylococcus aureus* and *Staphylococcus agnetis* exposed to eBeam at 8, 9, and 10 kGy and treated with formalin in preparation for this study (Figure 3). The BacTiter-Glo™ Microbial Cell Viability Assay (Promega, Madison, WI, USA) was utilized, and the ATP and O_2_ in bacterial cells were quantified in terms of relative luminescence, which is a direct indication of cells’ metabolically active and viable status.

The viability of 8 kGy-eBeam-treated *S. agnetis* was significantly higher than that of 10 kGy-treated cells during the week of vaccination, while the viability of eBeam-treated cells (all three doses) was significantly higher than the viability of formalin-treated cells in both strains. The assay was conducted weekly for 5 weeks following inactivation. Even after 5 weeks of inactivation, the viability of eBeam-treated cells (both strains) was similar between each dose but significantly different from that of formalin-treated *Staphylococcus*. This supported the selection of 8 kGy as the target eBeam dose for the present vaccine. Furthermore, the data suggest that the viability and metabolic activity of eBeam-treated cells are significantly higher than those of formalin-treated cells. Accordingly, despite their inability to replicate, eBeam-treated cells function as live cells, which is important for host immune response development. Therefore, this evidence supports the claim by Praveen et al. (2021) [38] that an eBeam vaccine is as efficient as a live vaccine but as safe as a killed vaccine.

**Figure 4:**
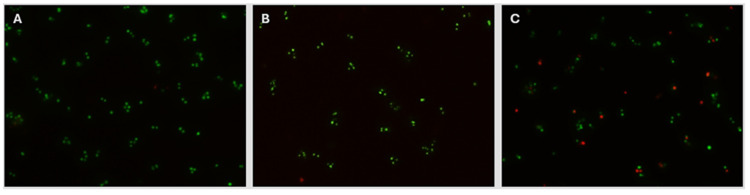
eBeam-treated cells retained their membrane integrity. Fluorescence microscopy images (100×) of *Staphylococcus* cells with (**A**) no treatment, (**B**) treated with 8 kGy-eBeam inactivation, and (**C**) treated with formalin inactivation, after being stained with the LIVE/DEAD BacLight Bacterial Viability Kit (Invitrogen Inc., Waltham, MA, USA).

The decision to select 8 kGy as the target eBeam dose for the present *Staphylococcus* vaccine was further supported by the evidence that the membrane integrity of eBeam-treated cells at 8 kGy was similar to that of non-treated cells. The BacLight assay (Invitrogen Inc., Waltham, MA, USA) was used, which utilizes a mixture of two fluorescent stains to identify cells with damaged membranes apart from those with intact membranes. Live cells with non-damaged membranes fluoresce green under fluorescent microscopy, while dead cells with damaged membranes fluoresce red. Figure 4 illustrates that the membrane integrity of non-treated and 8 kGy-eBeam-treated *Staphylococcus* cells was conserved, by the presence of a majority of green (live) cells. In contrast, the membranes of formalin-treated *Staphylococcus* cells were damaged, resulting in considerably higher numbers of red cells.

### 3.2. eBeam-Inactivated Staphylococcus Vaccine Significantly Decreased Lameness Compared to Other Treatments

**Figure 5:**
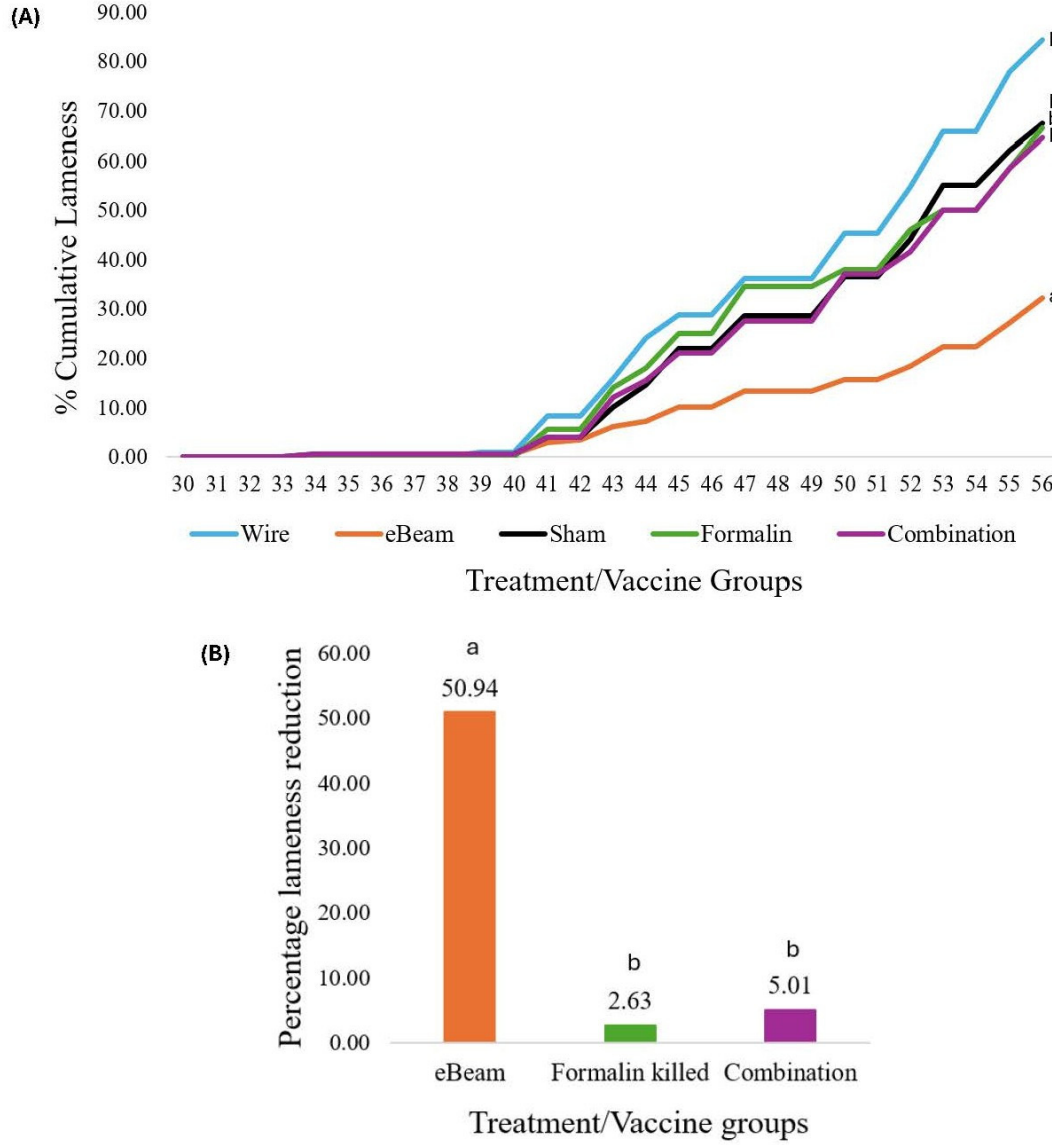
eBeam-inactivated *Staphylococcus* vaccine significantly reduced lameness. (**A**) Graph of variation in daily cumulative lameness of treatment groups (wire floor, eBeam, sham, formalin, and combination) through days 30–56. (**B**) Percentage lameness reduction in vaccine groups (eBeam, formalin, and combination) relative to sham group. a, b: Different letters denote significant differences between treatments (*p* < 0.05).

The daily cumulative BCO lameness was evaluated for all birds from D22 to the end of the trial. By D56, the total cumulative lameness of the WF group was 84.26%, while the control (sham) group had 67.5% cumulative lameness (Figure 5A), which illustrates the success of the wire-floor pens in inducing high lameness in birds and the effectiveness of the aerosol transmission model in disseminating lameness to the litter-floor pens. By D56, the daily cumulative lameness rates of the eBeam, formalin, and combination groups were 32.22%, 66.5%, and 64.5%, respectively (Figure 5A).

**Figure 6:**
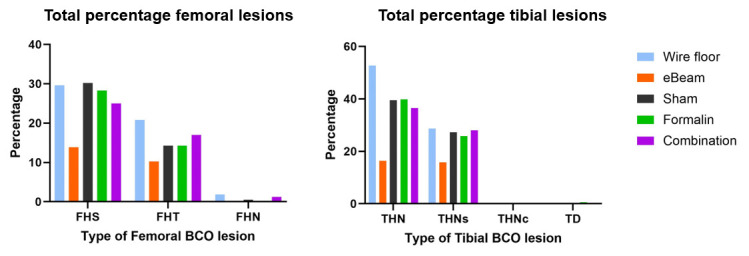
eBeam-vaccinated birds had fewer BCO lesions. Percentages of total femoral BCO lesions (FHS: Femoral Head Separation; FHT: Femoral Head Transitional degeneration; FHN: Femoral Head Necrosis) and total tibial BCO lesions (THN: Tibial Head Necrosis; THNs: Tibial Head Necrosis severe; THNc: Tibial Head Necrosis caseous; TD: Tibial Dyschondroplasia) in lame birds across treatment groups.

The birds that received the eBeam-inactivated *Staphylococcus* vaccine had a significant 50.94% reduction (*p* < 0.05) in daily cumulative lameness compared to the birds that received the sham vaccine and were raised on a litter floor (Figure 5B). The reduction in lameness in birds that received formalin-inactivated and combination vaccines was not significant (Table S1) compared to that in controls. Moreover, statistically non-significant reductions in total femoral and tibial BCO lesions were recorded in lame birds of the eBeam group compared to the others (Figure 6).

Since the eBeam-inactivated multi-strain-*Staphylococcus*-vaccinated group presented significantly a higher reduction in lameness and fewer BCO lesions compared to the other treatment groups, our results confirm the suitability of including *Staphylococcus* in the development of a vaccine against lameness, as it is among the most abundant causative agents of the disease.

### 3.3. Staphylococcus Was Absent in the BCO Lesions of Birds Vaccinated with the eBeam-Treated Staphylococcus Vaccine

**Figure 7:**
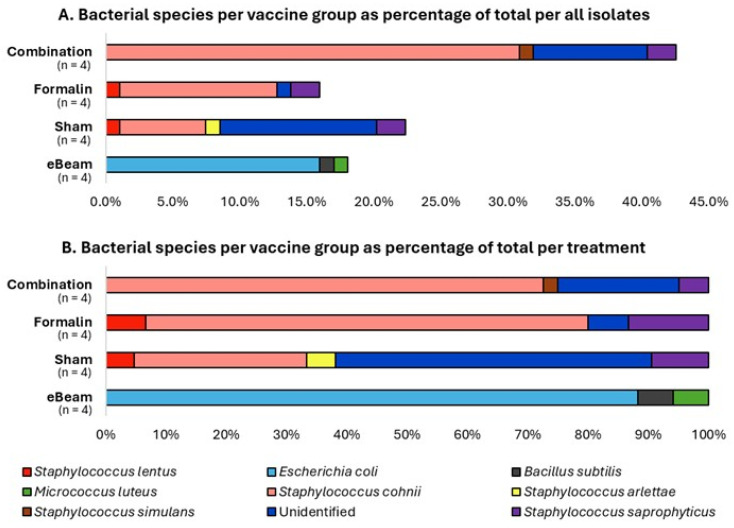
*Staphylococcus* was not identified in BCO lesions of eBeam-vaccinated birds. Percentages of bacterial species ((**A**) per total isolates and (**B**) per treatment) identified from the BCO lesions of lame birds across treatment groups (eBeam, sham, formalin, and combination). n represents the number of birds sampled.

In the present study, the bacterial species recorded with the highest incidence (48.94%) in the BCO lesions of lame birds was *Staphylococcus cohnii*, followed by *Escherichia coli* (15.96%) and *Staphylococcus saprophyticus* (6.38%) (Figure 7A). These results are in agreement with previously obtained evidence from several studies [11,54] that *Staphylococcus* spp. and *Escherichia coli* are among the most frequently associated bacteria with the pathology of BCO. Additionally, the unidentified species mentioned in Figure 7 are the sum of the bacteria that initially grew on Agar plates from the BCO lesion swab samples but did not re-grow during the process of re-culturing for isolating pure cultures, plus a minority of pure-culture isolates whose DNA extraction and sequencing were unsuccessful. Most importantly, the bacterial species isolated from the lame birds of the eBeam vaccine group did not contain *Staphylococcus* spp., unlike those from the other treatments (Figure 7B). This further confirms that the eBeam vaccine not only significantly reduced the total cumulative lameness but also effectively protected the host against the vaccine-targeted bacteria. This may be a reason behind the significant reduction in lameness, as *Staphylococcus cohnii*, the most abundant species in the present study, was successfully controlled in the eBeam vaccine group of birds.

### 3.4. Birds of the eBeam and Combination Vaccine Groups Had Higher Mucosal IgA Levels on D16 Compared to Other Groups, Suggesting Early Protection

**Figure 8:**
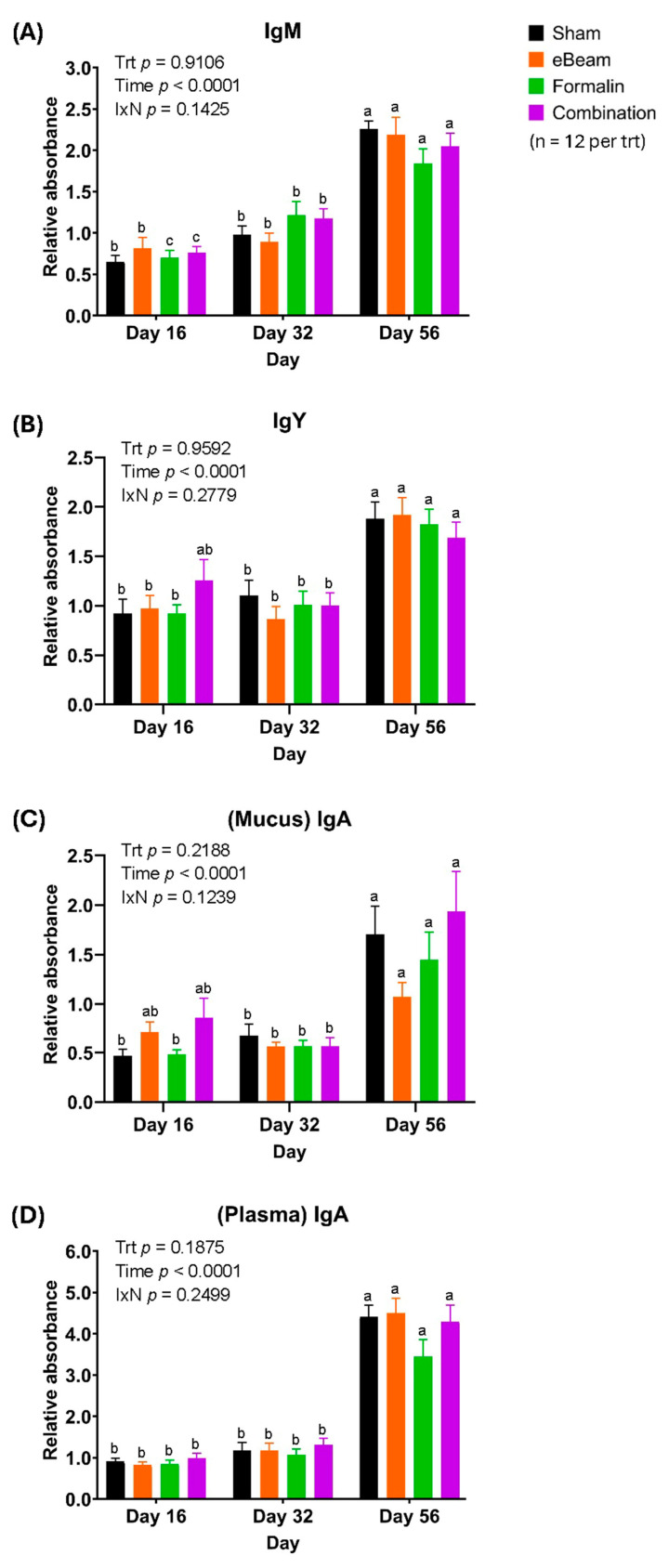
*Staphylococcus*-specific antibody levels changed similarly over time, increasing gradually in all treatments. Differences in blood plasma IgM (**A**), blood plasma IgY (**B**), tracheal mucous IgA (**C**), and blood plasma IgA (**D**) levels between treatment groups (sham, eBeam, formalin, and combination) on days 16, 32, and 56 of this study. Results are presented as means ± SEMs. a–c: Means without a common letter within a treatment group are significantly different (*p* < 0.05). Abbreviations: n—number of birds sampled per time point; trt—treatment (vaccine group); IxN—interactions (time × trt).

The time-course of circulating levels of IgM, IgY, and IgA, as well as mucosal IgA, is illustrated for each treatment group in Figure 8 below.

While the relative levels of IgM were not significantly different between treatments at any particular time point, there was a general trend of a significant increase in IgM levels over time within all treatments. Despite changes within the early time points, D56 exhibited significantly higher IgM levels in all treatments. However, the increase in blood IgM titers from D16 to D32 was significant in the formalin and combination vaccine groups as opposed to the other treatments (Figure 8A). The time-course of blood IgY profiles (Figure 8B) closely resembled that of IgM, with no significant differences between treatments at any particular time point but with significant increments within treatments over time. However, the difference in IgY levels between D16 and D32 of any treatment was not significant. The eBeam and combination vaccine groups showed numerically higher levels of IgA in tracheal mucus on D16 compared to D32 and statistically similar levels to those of D56 (Figure 8C). In contrast, the formalin- and sham-vaccinated birds exhibited lower levels of IgA in tracheal mucus on D16 and D32 than those on D56 (Figure 8C). Plasma IgA levels depicted no significant change between treatments at any particular time point (Figure 8D). The above general trend in the change in antibody levels (of all isotypes) may be explained by the fact that all birds were continuously exposed to the BCO-causing pathogens (including *Staphylococcus*) via aerosol. With an increase in the overall cumulative lameness of birds in the facility over time, pathogen doses correspondingly increased, resulting in higher loads of pathogens entering the respiratory tract and crossing into the bloodstream. In response, the natural immune response may have caused an elevation in antibody levels by D56 to combat the bacteria. Importantly, with exposure to the vaccine in the embryonic stage, the B lymphocytes of effectively vaccinated birds were expected to undergo affinity maturation and isotype switching to IgY and IgA isotypes. The eBeam and combination vaccine groups had higher mucosal IgA levels on D16 compared to the formalin and sham groups, which may have been due to being immunized with antigens of higher quality (with undamaged epitopes) from the former vaccines. This may have caused isotype switching to happen sooner, which in turn would increase in response to incoming pathogens early on. Furthermore, as BCO pathogens infected birds via aerosol in the present model, mucosal IgA of the respiratory tract would be the primary antibody to combat the invading pathogen. Higher mucosal IgA levels early in the time-course suggested better protection starting at an early stage via the eBeam and combination vaccines.

### 3.5. Leucocyte Populations Showed Diverse Trends of Concentration Changes Between Treatment Groups

**Figure 9:**
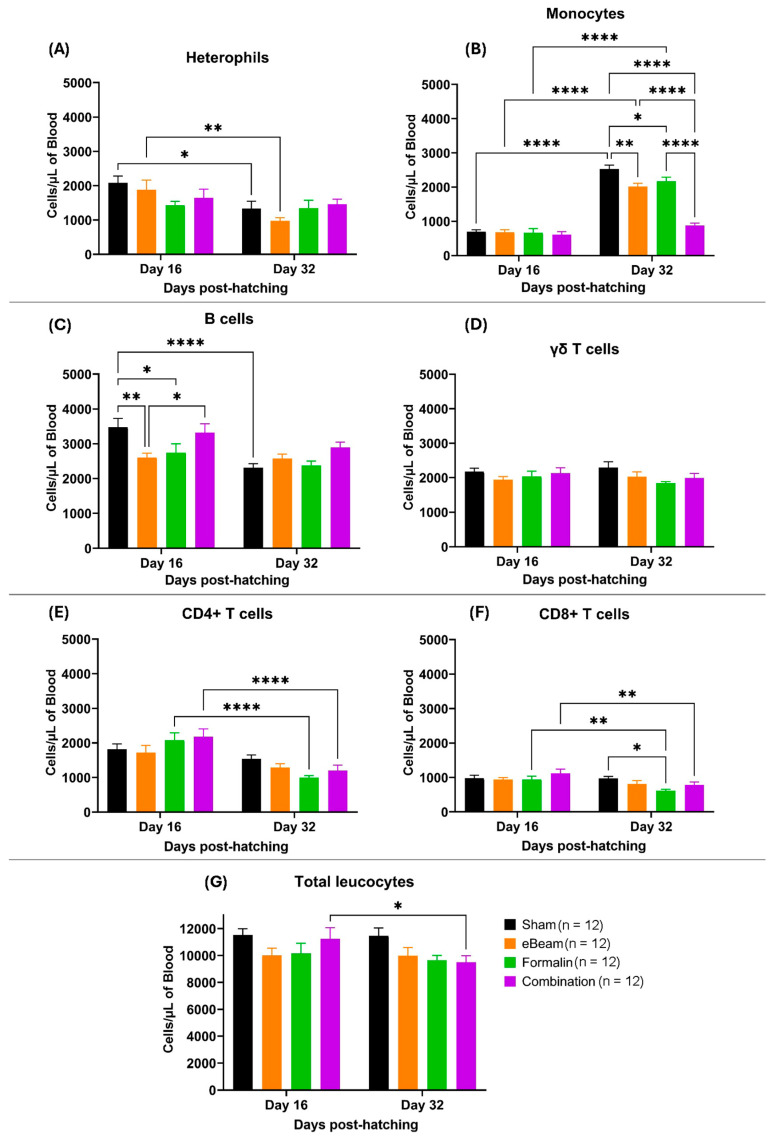
Concentration (cells/µL of blood) of heterophils (**A**), monocytes (**B**), B lymphocytes (**C**), γδ T lymphocytes (**D**), CD4^+^ T lymphocytes (**E**), CD8^+^ T lymphocytes (**F**), and total leucocytes (**G**) over sampling days (16 and 32) in differently vaccinated groups of broilers (sham, eBeam, formalin, and combination). Graphs show mean ± SEM. *p* value: * ≤ 0.05; ** ≤ 0.01; **** ≤ 0.0001. n represents number of birds sampled per time point.

Blood cell population levels exhibited significant temporal variation from before BCO clinical symptoms (D16) to when birds typically began to show symptoms of lameness (D32). The eBeam group exhibited the most significant drop in heterophil levels (cells/µL) from D16 to D32 (Figure 9A). Monocytes showed a significant increase from D16 to D32 in the sham, eBeam, and formalin vaccine groups (Figure 9B), which may have been due to increased production to combat the invading pathogen. However, the increase in monocyte levels from D16 to 32 in the combination group was not significant, which may have been due to either lower monocyte production in the combination-vaccinated birds or early recruitment from the blood to the site of infection. The eBeam group showed significantly lower levels of B cells on day 16 compared to other treatments. B cells showed a general trend of decreasing from D16 to D32 in all treatments, except in the eBeam vaccine group (Figure 9C). γδ T lymphocytes (Figure 9D) and total leucocytes (Figure 9G) showed almost no significant variation over time in all treatments. Significant reductions in CD4^+^ (Figure 9E) and CD8^+^ T lymphocyte (Figure 9F) levels from D16 to D32 were prominent in the formalin and combination vaccine groups. Overall, all four treatment groups presented similar blood cell population distributions at both time points. However, there was a significant increase in monocytes in the bloodstream of the birds between days 16 and 32, suggesting an immune response against ongoing infection.

## 4. Discussion

Bacterial Chondronecrosis with Osteomyelitis (BCO) is a primary concern currently faced by the poultry industry [55,56]. While the etiology and pathogenesis of the disease are incompletely understood [21,22,57], it is known to be of opportunistic bacterial origin and remains the leading cause of lameness [58,59] in broiler chickens, evidently warranting investigation into control and prevention. The aerosol transmission model developed by our lab has been proven successful in assessing treatment effects in the prevention of lameness. This success greatly lies in the model’s ability to rapidly induce lameness in the birds raised on wire floor, followed by successful transfer to the birds raised on litter floor. The present study achieved approximately 85% lameness (percentage of lame birds among the total birds) in wire-floor pens, followed by approximately 68% lameness in litter-floor pens of the control (sham) group. This demonstrates a numerical increment of roughly 25% lameness in wire-floor pens compared to the sham pens, representing the success of the infection source. Simultaneously, the difference was statistically insignificant (*p* > 0.05), indicating the success of lameness transfer by the current model, as demonstrated by previous trials in our lab [20,49,55,60].

Amidst an established stressful, lameness-stimulatory environment, the birds vaccinated with eBeam-inactivated *Staphylococcus* achieved a significant reduction of approximately 51% in lameness compared to the sham group. In contrast, the formalin and combination vaccine groups presented low lameness reductions, with a similar prevalence of the disease as that of the control group. This success may be attributable to the retention of membrane integrity [18,34,37] and viability [38] of eBeam-inactivated bacteria, symbolizing a live vaccine, as opposed to cross-linking of membrane proteins [61,62,63,64] causing significant loss of membrane integrity (Figure 4) and viability (Figure 3) in formalin-inactivated bacteria, favoring a better immune response against *Staphylococcus* in birds vaccinated with the eBeam-inactivated *Staphylococcus* vaccine. Moreover, the present study surpassed the lameness reduction percentages reported in other studies [16,65,66] utilizing feed additives. In addition to the success in reducing lameness, the cost and labor efficiency (one-time in ovo vaccination versus mixing feed additives with the diet and feeding birds over the entire production period) of the eBeam-inactivated vaccine makes it a potential alternative to feed additives. According to the current understanding of the pathogenicity of BCO, the frequent causative agents among a variety of other bacterial species are *Staphylococcus* spp., *Escherichia coli*, and *Enterococcus* spp. [11,20,54,67,68,69]. This trend is represented in the present study through the identification of *Staphylococcus cohnii* as the most abundant species (approximately 49%), followed by *Escherichia coli* (approximately 16%) as the second-most abundant bacterial species (Figure 7), isolated from the BCO lesions of lame birds. Interestingly though, birds of other treatment groups (except eBeam) contributed to the abundant discovery of *S. cohnii* in BCO lesions, alongside other *Staphylococcus* species (*S. simulans*, *S. arlettae*, *S. saprophyticus*, and *S. lentus*). eBeam-vaccinated birds showed *Escherichia coli* in high abundance alongside *Micrococcus luteus* and *Bacillus subtilis* isolated in smaller percentages. This certainly demonstrates the success of the eBeam vaccine in restricting *Staphylococcus cohnii* colonization of BCO lesions and suggests possible cross-protection against other *Staphylococcus* spp., which were neither included in the vaccine nor detected in birds of the eBeam group but were present in birds of the other groups. Furthermore, the fact that *Staphylococcus* dominated BCO lesions of the rest of the birds, as opposed to *E. coli*’s domination in the eBeam-inactivated *Staphylococcus*-vaccinated birds, suggests the possible over-competition of *Staphylococcus* over *E. coli*. However, this also provides insight into the possibility that the inclusion of more bacterial species associated with the pathogenesis of BCO in an eBeam-inactivated vaccine would give even greater protection against a broad range of pathogens. However, the presence of a minority of unidentified species of bacteria (due to failed isolation and re-culturing or failed DNA amplification and sequencing) was a limitation, as there may have been other bacterial species within the BCO lesions of birds of the combination, formalin, and sham groups. Yet this does not significantly impact the present study’s conclusion as all the bacterial species isolated from the BCO lesions of birds of the eBeam group were identified.

Based on previous experience, the visual onset of clinical lameness in broilers was initiated around D25–D35 [12,49,55,56,60,70] of the experimental model, and it occurred on D34 in the present study. The sampling time points were decided to cover periods before, during, and after the onset of visual clinical lameness. Besides the overall fluctuations in IgM (Figure 8A), IgY (Figure 8B), and IgA (Figure 8C,D) across the sampling time points in this study, no significant differences between treatment groups were observed at any given time point. A possible explanation may be the absence of an actual ‘negative’ control in this study design, which did not include a group of birds entirely unexposed to the pathogen. Hence, a true background (control) level of antibodies to compare the treatment responses was not identified. The differences obtained may have been significant if compared with a truly negative population. This warrants future studies to include birds unexposed to infection, to circumvent this limitation and obtain baseline antibody levels for a wholistic interpretation of immune responses. Yet the eBeam vaccine’s success in significantly reducing lameness compared to all other groups and the absence of *Staphylococcus* in BCO lesions of birds receiving the eBeam vaccine is undeniable. Despite the relative levels of antibody production by the eBeam vaccine being similar to that of other groups, our results suggest that the antibodies produced by eBeam-vaccinated birds were more efficient at preventing bacterial translocation, possibly due to their specificity to better-suited and/or a greater variety of epitopes to eliminate the bacteria effectively. This could potentially enhance the effectiveness of IgA’s localized role, i.e., providing the first line of defense in eliminating inhaled *Staphylococcus* in the respiratory tracts, more efficiently in the eBeam-vaccinated birds. It could be argued that the greater lameness reduction observed in the eBeam-vaccinated birds was due to a more effective stimulation of cell-mediated immunity, targeting intracellular *Staphylococcus* at the site of infection. This, in addition to the observed high-quality, *Staphylococcus*-specific antibody production, would support more efficient and targeted bacterial clearance and protection from further infection. Although the combination vaccine group showed similar mucosal IgA and IgY responses to the eBeam vaccine, the overall protection in terms of lameness reduction was significantly lower. A possible explanation is that the combination vaccine was prepared with half of the dose of each vaccine, which may not have been concentrated enough to generate the effective response seen with the full dose of the eBeam vaccine alone. Furthermore, despite having similar circulating CD4+ T cell levels in the birds of eBeam vs. other groups on D16 and D32, the optimum Th1/Th2 balance [71] of the eBeam group may have resulted in a balanced cell-mediated and humoral immune response, effectively eliminating *Staphylococcus* and reducing lameness.

Leucocyte and antibody recruitment in response to cytokines and chemokines released by infected tissues typically occurs within hours of the onset of infection [72,73]. Furthermore, significant increases in plasma antibody profiles in eBeam-vaccinated birds compared to controls have been recorded when sampled at shorter time intervals (<16 days) between challenge and sample collection for other bacterial species [18,34]. Moreover, other studies showed that by the 10th day post-challenge, significant *Staphylococcus aureus*-mediated cytomorphological changes in thymus, liver, and spleen tissues were recorded in control-group chickens, compared to those that received treatments against *Staphylococcus aureus* [74]. Based on these findings, significant differences in antibody and blood cell population profiles between the eBeam group and other groups may have been detected prior to day 16 in the present study. Clarification of this requires further research, and it would be advisable to sample blood and tracheal mucus at earlier time points in future studies, as the onset of infection in the aerosol transmission model is inconspicuous (as opposed to providing bacterial challenge via oral gavage, drinking water, etc.). Identifying immune responses against *Staphylococcus* can be challenging due to its numerous adaptations to evade and misguide host immune responses. For example, their polysaccharide capsules interfere with complement-mediated phagocytosis by masking the antigens to which host-complement factors are bound [25]. Furthermore, certain strains possess anti-phagocytic properties [26] and produce exotoxins that act as superantigens, resulting in the polyclonal activation of T cells (independent of antigen specificity) and the release of proinflammatory cytokines by T lymphocytes, thereby rendering the host vulnerable and misleading their immune system [27]. Protein A produced by *S. aureus* prevents antibody-mediated phagocytosis in mammals, by binding to the Fc regions of IgG, thereby preventing Fc-receptor-mediated phagocytosis of antibody-opsonized antigen [28], although it should be noted that in chickens, *S. aureus* protein A does not bind to IgY (chicken IgG-like immunoglobulin isotype) [75].

Additionally, the D_10_ value is the dose of radiation required to achieve a 90% reduction in a microbial population [76,77]. Recent studies have demonstrated that the D_10_ value for inactivating *Staphylococcus* with eBeam and gamma ranges from 0.5 kGy–1 kGy [78,79,80,81,82]. A further understanding of the D_10_ values of multiple *Staphylococcus* strains would assist in the precise optimization of the eBeam inactivation dose, thereby minimizing damage to membrane epitopes and maximizing viability while achieving non-reproducibility, which would increase the protection of the vaccine. Expanding this knowledge to other bacterial genera involved in the pathogenesis of BCO would facilitate the production of an eBeam-inactivated vaccine containing multiple genera of BCO-causative bacteria, which may provide better protection against BCO.

## 5. Conclusions

Bacterial Chondronecrosis with Osteomyelitis is currently among the leading causes of lameness in broiler chickens, resulting in reduced animal welfare and significant financial losses to the poultry industry. This condition is frequently driven by multiple bacterial species and mainly by *Staphylococcus* species infection, which leads to the necrosis of long leg bones. The electron beam-inactivated, multi-strain *Staphylococcus* vaccine tested in the present study significantly reduced BCO lameness in broiler chickens and successfully reduced *Staphylococcus* colonization of BCO lesions on the femurs and tibiae. This study provides insight into possible means of achieving stronger protection against BCO lameness via further optimization of the vaccine.

## 6. Patents

University of Arkansas: Patent Pending # PCT/US25/34205: “Electron Beam (eBeam)-Killed Multi-Bacterial Vaccines”.

## Figures and Tables

**Table 1:** Treatment groups of this vaccine study.

Treatment	Flooring	Vaccine	Number of Pens Allocated
WF—Infection source ^1^	Wire	sham	2
eB—eBeam group ^2^	Litter	eBeam-inactivated	4
FK—Formalin group ^3^	Litter	formalin-inactivated	4
F+E—Combination group ^4^	Litter	eBeam + formalin	4
Sham—Control group ^5^	Litter	sham	4

^1^ Birds raised on a wire floor develop the lameness disease at an accelerated rate due to stress, and the house design transmits the pathogens via aerosol to all other pens (infection source). ^2^ Birds vaccinated with 10^7^ CFU/mL of the electron beam-inactivated vaccine. ^3^ Birds vaccinated with 10^7^ CFU/mL of the formalin-inactivated vaccine. ^4^ Birds vaccinated with a combination of the electron beam- and formalin-inactivated vaccines. ^5^ Birds vaccinated with pure culture media (Tryptic Soy Broth) without bacteria.

## Data Availability

Raw data supporting the conclusions of this manuscript will be available upon request.

## Supplementary Materials

The following supporting information can be downloaded at: https://www.mdpi.com/article/10.3390/vaccines13090946/s1, Table S1. Statistical comparison of percentage lameness reduction (*p* values and standard error) between treatment groups (Wire floor, eBeam, Formalin, Combination and Sham) based on logistic regression, utilizing a Generalized Linear Model in R version 4.2.2.
