# Supplementary material for: Leveraging Electron Beam-Inactivated Multi-Strain Staphylococcus Vaccine for Preventing BCO Lameness in Broiler Chickens
Source: Vaccines (Basel). 2025 Sep 4;13(9):946. doi: 10.3390/vaccines13090946 (PMC12474430; doi:10.3390/vaccines13090946)
Supplement: Supplementary file 1 [file vaccines-13-00946-s001.zip › vaccines-3832129-supplementary.pdf]

Table S1. Statistical comparison of percentage lameness reduction (*P* values and standard error) between treatment groups (Wire floor, eBeam, Formalin, Combination and Sham) based on logistic regression, utilizing a Generalized Linear Model in R version 4.2.2.

	eBeam		Sham		Formalin		Combination	
	***P* value**	**Std. error**	***P* value**	**Std. error**	***P* value**	**Std. error**	***P* value**	**Std. error**
**Wire floor**	0.00213	0.35494	0.53187	0.341711	0.63999	0.348158	0.50921	0.341651
**eBeam**			0.00315	0.296923	0.00206	0.300968	0.00388	0.299508
**Sham**					0.85906	0.286011	0.96664	0.284312
**Formalin**							0.8283	0.288985
